# Geographical distribution and access of burn victims to a specialized treatment unit: a cross-sectional study

**DOI:** 10.1590/acb407425

**Published:** 2025-10-17

**Authors:** Wilson Falco, Pedro Henrique Domingos, Gabriel Sanchez Okida, João Felipe Pissolito, Pedro Henrique Soubhia Sanches, Lucas Ribeiro de Azevedo, Marcelo Oliveira Mourão, José Antônio Sanches, Alfredo Gragnani

**Affiliations:** 1 Centro Universitário Padre Albino – Faculdade de Medicina de Catanduva – Catanduva (SP) – Brazil.; 2 Universidade Federal de São Paulo – Departamento de Cirurgia Plástica – São Paulo (SP) – Brazil.

**Keywords:** Epidemiology, Burns, Brazil

## Abstract

**Purpose:**

To analyze the geographical distribution of patients treated at a burn treatment unit (BTU) in Catanduva, São Paulo, Brazil, and examine the relationship between geographic and clinical variables.

**Methods:**

This is a cross-sectional study that analyzed patients hospitalized for burns between January 2018 and May 2022. Data were obtained from medical records and included patients’ residence city, age, and percentage of total body surface area burned (%TBSA). The data were processed using QGIS and R, and travel distances and times were calculated. Statistical analyses included bivariate and correlation tests.

**Results:**

The total of 1,164 patients were analyzed. Most of them resided outside Catanduva, totaling 277 cities. The average distance was 179.37 km, and the average travel time was 140.94 minutes. Patients from Catanduva had an average age of 35.55 years old, and the average %TBSA was 12.15. Patients from outside Catanduva were significantly younger and had a higher %TBSA than the local patients. A weak but significant negative correlation was found between patient age and distance to the BTU (ρ = -0.14, *p* < 0.05), while %TBSA showed a weak positive correlation with travel distance (ρ = 0.21, *p* < 0.05). No significant differences were observed between the pre- and pandemic periods.

**Conclusion:**

This study highlights regional differences in specialized burn care access and may inform policy aimed at reducing care disparities.

## Introduction

Burns are a universal health concern, with an estimated 180,000 deaths per year caused by this kind of injuries. The countries most affected are low- and middle-income countries. In addition, non-fatal burns are a considerable cause of morbidity^1^. In Brazil, domestic accidents are the main cause of burns, with around one million cases recorded annually, according to the Ministry of Health^2^^,^ 3. According to data from the December 2022 Epidemiological Bulletin, between 2015 and 2020, 19,772 deaths from burns were recorded in the country, more than half of which were due to thermal incidents. In addition, data from the Unified Health System shows that this condition is responsible for around 30,000 hospital admissions a year^3^^,^ 4.

Burn treatment units (BTU) aim to improve care for patients who have suffered burns, helping to improve the epidemiological indices of this type of care^5^. While there are still few studies on access to BTUs in Brazil, international research has explored relevant aspects of this issue.

Klein et al.^6^, for example, evaluated state, regional, and national access to burn centers in the United States of America, considering air and ground transportation, and observed significant inequalities between regions. In a complementary way, Edwards et al.^7^ analyzed transport costs and access to the nearest burn center, highlighting regional disparities and higher costs in the rural population.

Daher et al.^8^, in Brazil, showed that there are areas with difficulties in accessing BTUs, with the concentration of these specialized centers predominantly in the southeast region. This evaluation is fundamental, as it is directly related to the costs of the public health system and care provided to critically ill patients.

Despite this analysis, there is a lack of studies that assess access to BTUs in detail and the origin of patients treated at each unit. This evaluation is fundamental, as it is directly related to the costs of the public health system and the quality of care provided to critically ill patients. Given this context, the present study aimed to investigate whether geographical distance and travel time to a specialized BTU are associated with patient demographics and clinical severity, thereby assessing potential disparities in access to care, and also, using data from the cities where which patients live, to investigate the geographical distribution of patients of the Padre Albino Hospital BTU, in Catanduva (SP), Brazil, and explore the association with aspects of its clinical variables in this cohort. In addition, we checked whether there were any changes in distance or time during the COVID-19 period.

## Methods

This study was a secondary analysis of a cross-sectional observational study. Data from burn injury patients hospitalized between January 2018 and May 2022 were analyzed using medical records from the BTU of Catanduva as the primary source. De-identified data were used throughout the entire process, and the study was approved by the Research Ethics Committee (CAAE: 62621822.50000.5430). Detailed methodology has been described in a previous publication^9^. Data supporting the findings of this study are available upon request from the corresponding author.

The subgroup classification followed those of the original study, categorizing individuals as “children and adolescents” (0 to 18 years old) and “adults and elderly” (over 18 years old). All patients treated during the period analyzed were considered eligible and were included in this study. Only cases with incomplete information in the medical records were excluded. The inclusion criteria were:

- Patients of all ages (children, adolescents, adults, and elderly);
- Admission to the BTU in the specified study period;
- Availability of complete data on city of residence, age, and percentage of total body surface area burned (%TBSA).

Patients were excluded if: medical records were incomplete, specifically lacking data on age, %TBSA, or place of residence; the patients had burns, specifically those diagnosed with Stevens-Johnson syndrome or epidermal necrolysis. The total was three cases.

The temporal division of the initial study was also maintained, considering the COVID-19 pandemic. The periods were defined as “pre-pandemic” (May 2018 to February 2020) and “during the pandemic” (March 2020 to December 2021). For analytical purposes, patients were also categorized based on their residence as local (residents of Catanduva) or external (residents of other cities). The analyzed variables included percentage of total body surface area burned (%TBSA) and patient age (years old).

The data regarding the cities of origin refer to the patients’ place of residence and not necessarily the location of the initial treatment. The names of all the cities were manually verified and corrected according to the Brazilian Institute of Geography and Statistics (IBGE)^10^. Once the database was prepared, the data were processed using QGIS software and linked to an IBGE city map, from which the coordinates of each city’s center were obtained^10^^,^ 11. The same software was used to create descriptive maps.

**Figure 1:**
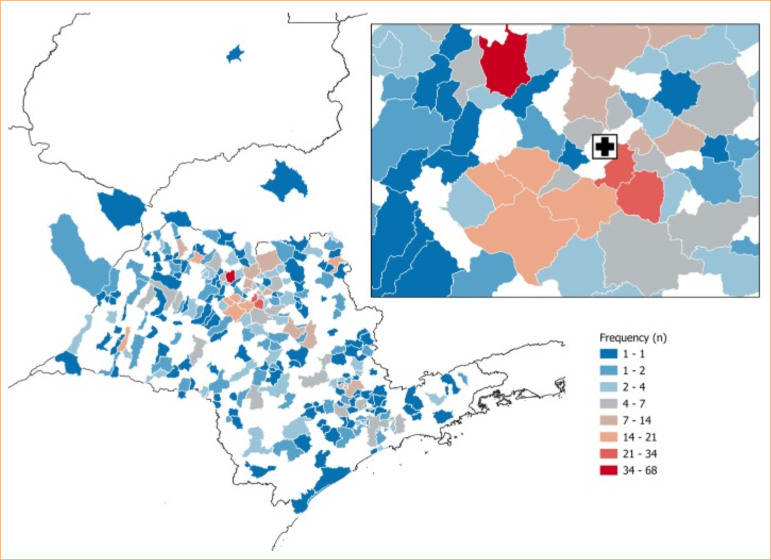
Geospatial distribution of patients’ cities of residence.

Source: Elaborated by the authors.

**Figure 2:**
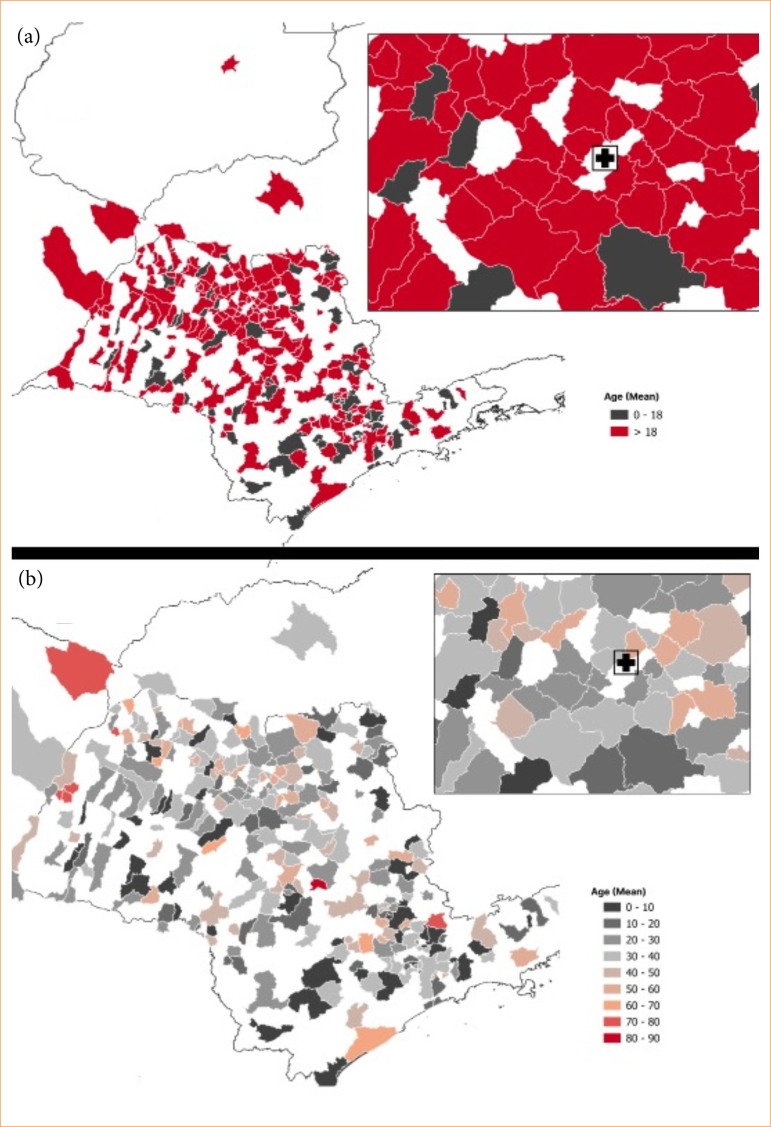
Geospatial description: average age in each city of residence of patients. **(a)** Geospatial distribution of the mean age of patients in each municipality of residence, showing that areas farther from Catanduva, SP, Brazil, predominantly present a mean age below 18 years old. **(b)** Enlarged view of the region adjacent to Catanduva, highlighting municipalities with a mean age greater than 20 years old.

Source: Elaborated by the authors.

**Figure 3:**
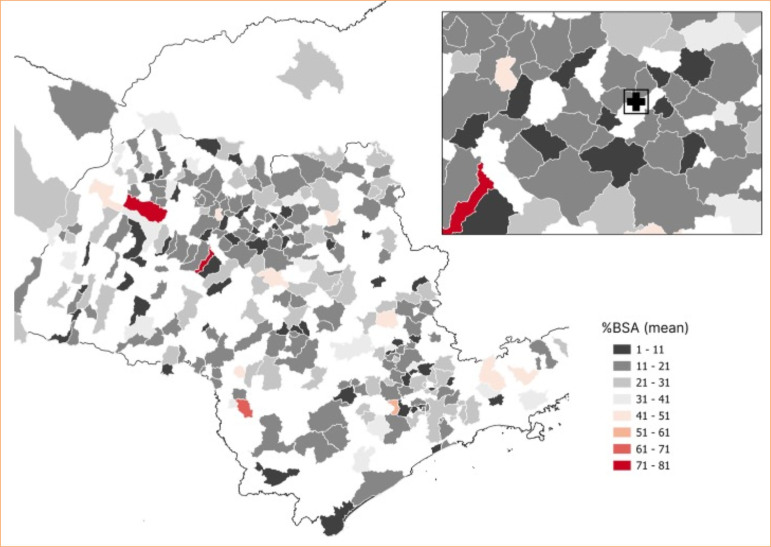
Geospatial description: average % total body surface area burned (BSA) in each city of origin of patients.

Source: Elaborated by the authors.

Once the city centers were obtained, the R Studio software was used^12^. Using the open-source routing machine^13^ package, the distance and duration of routes between the centers of the origin and destination cities were calculated. This technique uses maps and, through Dijkstra’s algorithm, determines the distance between points, considers road networks, and estimates the travel time (in minutes) by car. In all maps, Catanduva city is highlighted with a cross inside a rectangle (Figs. 1–3).

Using R Studio software, bivariate analyses (analysis of variance and T-tests with Bootstrap) were performed, verifying assumptions (homogeneity of variance and normality) and applying corrections when necessary. Spearman’s correlation analyses were also performed. All tests were considered statistically significant at *p* < 0.05^12^. Standard deviation (SD), confidence interval (95%CI), and standard error (SE) were reported when necessary.

## Results

The total of 1,164 patients were analyzed. Among them, 268 were from the same city as the BTU, Catanduva. In addition to patients from the city itself, 896 patients from 277 different cities were evaluated. The city with the highest number of patients was São José do Rio Preto (7.59%), followed by Pindorama (3.79%), and Santa Adélia (3.35%), which are cities near Catanduva (identified with a cross on the maps). In general, a distribution was observed throughout São Paulo state, with some patients coming from the states of Minas Gerais, Goiás, and Mato Grosso do Sul. The highest frequency of patients was found in cities close to the hospital (Fig. 1).

The first analysis conducted focused on comparing clinical aspects and the distance between external patients and those from Catanduva (Fig. 1). Among patients from other cities, the average age was 30.14 years old (SD = 21.09), and the mean percentage of total body surface area burned (%TBSA) was 19.59% (SD = 14.01). When evaluating the travel distance, the observed values ranged from 13.03 to 625.03 km, with an average of 179.37 km (SD = 135.24). The average travel time from the city of residence to BTU was 140.94 minutes (SD = 100.87). None of the continuous variables showed normality in the Shapiro-Wilk’s test (*p* < 0.05).

Patients from Catanduva had an average age of 35.55 years old (SD = 20.82), and the average %TBSA was 12.15 (SD = 9.47). When comparing the means of age and %TBSA between local and external patients, a difference of 5.39 years (95%CI 2.41–8.08; SE = 1.45; *p* < 0.05) and 7.42% (95%CI -8.91–-5.87; SE = 0.74; *p* < 0.05), respectively, was observed.

The second analysis compared the periods (pre-pandemic versus during the pandemic). Similarly, no significant differences were observed between the groups. The average travel distance before the pandemic was 181.69 km (SD = 138.01), with an average travel time of 143.55 minutes (SD = 103.25). During the years evaluated (Table 1), the minimum average distance observed was 167.87 km (2018), while the maximum was 195.18 km (2019).

**Table 1:** Distance and travel time to the city of residence of patients.

	Distance (km)						Time (minutes)			
	n	Mean	SD	Median	*p* -value*		Mean	SD	Median	*p* -value*
External	896	179.37	135.24	147.13	-		140.94	100.87	120,5	-
Age groups (years old)										
> 18	614	164.57	129.73	119.62	< 0.01		130.22	97.32	105.5	< 0.01
0–18	282	211.59	141.49	221.61			164.26	104.64	173.1	
Sex										
Female	344	185.43	137.29	163.81	> 0.05		144.96	102.55	125.6	> 0.05
Male	552	175.59	133.93	147.13			138.43	99.82	111.7	
Pandemic**										
Before	478	181.69	138.01	162.44	> 0.05		143.55	103.25	125.6	> 0.05
During	352	177.16	132.06	147.13			138.55	98.39	112.95	
Year †										
2018	222	167.87	138.92	118.25	> 0.05		134.52	105.03	105.5	> 0.05
2019	223	195.18	137.72	185.59			152.44	102.23	153.3	
2020	221	180.69	131.35	162.75			140.42	97.83	123.2	
2021	165	173.03	132.11	133.95			136.64	98.51	111.2	
2022	65	175.99	133.61	147.13			136.06	97.45	120.5	

SD: standard deviation; Before: May 2018 to February 2020; During: March 2020 to December 2021;

† Year of patient admission;

* bivariate tests (T-tests with Bootstrap and analysis of variance);

** there are no missing data for these variables.

Source: Elaborated by the authors.

The third analysis aimed to investigate the geographic distribution according to clinical and sociodemographic aspects, including age subgroups, sex, and %TBSA (Table 1). When evaluating the age subgroups, the average distance for adult and elderly patients was 164.57 km (SD = 129.62), which was lower than that of children and adolescents, 211.59 km (SD = 141.49). A significant difference of -46.78 km (95%CI -65.58–-26.66; SE = 9.89; *p* < 0.01) was identified. The difference in travel duration was -34.14 minutes (95%CI -48.75–-19.41; SE = 7.34; *p* < 0.01).

The difference in distance between age subgroups was also evident in the maps. More distant locations from Catanduva showed patients with an average age below 18 years old, whereas cities closer to the BTU showed an average age above 18 (Fig. 2a). The cities immediately adjacent to Catanduva had an average age of > 20 years old (Fig. 2b). When evaluating the correlation between age and distance to BTU, a weak correlation was observed (ρ = -0.14, *p* < 0.05).

There was no significant difference between the sexes, although the average distance for female patients was slightly higher, at 185.43 km (SD = 137.29), with an average travel time of 144.96 min (SD = 102.55) (Table 1).

Regarding the %TBSA, there was a predominance of cities with average values between 11–21% and 21–31%. Cities immediately surrounding Catanduva had average %TBSA values of 11–21% and 1–11% (Fig. 3). A weak correlation was observed between the %TBSA and travel distance or duration (ρ = 0.21, *p* < 0.05).

## Discussion

This study analyzed the transportation of patients to the BTU in Catanduva, highlighting aspects such as distance, travel time, clinical characteristics, and the impact of the pandemic. Patients from São Paulo state were observed, and some out-of-state patients were included in the analysis. Excluding patients from Catanduva, the average travel time to BTU was over two hours, with an average distance of 179 km. Most patients were adults, while children and adolescents were more prevalent in cities farther from BTU. Analyzing hospitalizations from cities adjacent to Catanduva, the average patient age was > 20 years old. No significant differences were observed in sex. The average %TBSA was 12.15%. No significant differences were found between the pre- and pandemic periods.

Daher et al.^8^ results support the findings of this study, regarding regional inequalities in access to specialized burn treatment in Brazil. While the authors mapped population coverage and identified significant gaps, especially in remote areas outside the southeast region, this study provides a deeper analysis by exploring the individual trajectories of patients treated at a single specialized unit, integrating clinical and geographical variables.

However, the analysis revealed that even in a more restricted context regional inequalities persist. Patients from more distant cities face travel times exceeding six hours, particularly in pediatric subgroups. Although São Paulo has the highest concentration of specialized hospitals in Brazil, 23% of BTU patients in Catanduva came from distant cities across the state, with some even from outside São Paulo, indicating that access barriers remain evident even in regions with better infrastructure. These findings emphasize the urgency of broad strategies to expand coverage and reduce regional disparities in burn treatment.

An American study evaluated geographic access to burn centers in the country, revealing that only a minority of the population lives in a two-hour ground transport distance from a burn center verified by the American Burn Association^6^.

Similarly, the present study showed that the average travel duration from the patient’s city of residence to the evaluated BTU was 140.94 minutes (SD = 100.87). Notably, patients experiencing transport delays to burn centers tend to have longer hospital stays and higher infection rates, given the specialized nature of burn treatment, which requires rapid intervention to reduce complications and mortality^14^. Thus, regional transfer and transport protocols have proven effective in ensuring proper care even with prolonged transport times.

Additionally, when analyzing subgroups, this study found that the average distance for adults and elderly patients was 164.57 km, which was lower than the 211.59 km observed for children and adolescents, with a significant difference of -46.78 km. Similarly, a study by Edwards et al.^7^ also highlighted differences in travel distance between age subgroups, showing that pediatric patients tend to live farther from burn centers than adults, with higher associated transportation costs.

This study can also guide burn prevention interventions as it identifies cities with higher incidence rates, where reducing cases could directly impact BTU bed availability, such as prevention campaigns. Thus, local prevention strategies are crucial and require continuous improvement, considering that 23% of the 1,164 patients resided in Catanduva.

This study has limitations. First, as it uses data from patients’ cities of residence, it is not possible to infer that the accident occurred in the same location, although most burns are domestic^3^^,^ 4. Second, this was a single-center study. Third, this study did not assess clinical outcomes such as mortality, length of stay, or infection rates, which limits the analysis of potential consequences of delayed access. Similar future studies could provide valuable public health data, supporting the expansion of BTU beds, and multicenter studies could provide better insights into the impact of distance and travel time on important clinical outcomes.

## Conclusion

This study successfully described the geographical distribution of patients treated at a BTU and explored the association between travel distance and time to residence with clinical variables, providing valuable information for the Health Department. Despite observing differences in distance based on age subgroups, no significant differences were found in overall access patterns by sex or pandemic period. Future multicenter studies incorporating outcomes such as mortality and length of stay are essential to explore the impact of access barriers. In the meantime, health authorities should consider strategies to expand BTU coverage and optimize referral pathways, especially for pediatric patients in remote areas. This evidence can also inform prevention policies focused on high-incidence cities, supporting both early intervention and resource planning in the public health system. Further studies and multicenter collaborations are essential for advancing burn care research in Brazil.

## Data Availability

The data will be available upon request.
